# Impact of Gold Nanoparticles on Testosterone Metabolism in Human Liver Microsomes

**DOI:** 10.1186/s11671-019-3021-z

**Published:** 2019-06-17

**Authors:** Kyoungju Choi, Hyun Joo

**Affiliations:** Department of Anatomy & Physiology, Nanotechnology Innovation Center of Kansas State (NICKS), College of Veterinary Medicine, Kansas State University, Manhattan, KS 66506 USA

**Keywords:** Gold nanoparticles, Human plasma protein corona, Testosterone metabolism, Human liver microsomes, Cytochrome P450, Individual variation

## Abstract

Gold nanoparticle (AuNP)-protein corona complexes can alter cytochrome P450 (CYP)-mediated testosterone (TST) metabolism by altering their physicochemical properties. We investigated the impact of NP size, surface chemistry, and protein corona in TST metabolism in pooled human liver microsomes (pHLM) employing 40 and 80 nm AuNP functionalized with branched polyethylenimine (BPEI), lipoic acid (LA), and polyethylene glycol (PEG) as well as human plasma protein corona (PC). Individual variation in AuNP-mediated TST metabolism was also characterized among single donor HLM that contained different levels of CYP activities. Inhibitory effects of 40 nm AuNP and to a lesser degree of 80 nm AuNP occurred for the production of a total of five hydroxylated metabolites of TST in pHLM but PC alleviated them. Meanwhile, naked AuNP increased androstenedione production. Interindividual variation in TST metabolism occurred within single donor HLM. In most cases, 40 and 80 nm naked and PC AuNP essentially suppressed TST metabolism at non-inhibitory concentration but PC PEG-AuNP increased androstenedione. These studies contribute to a better understanding of the role of AuNP as TST disruptor by altering TST metabolism and could be utilized to screen other NP as potential endocrine disruptor.

**Electronic supplementary material:**

The online version of this article (10.1186/s11671-019-3021-z) contains supplementary material, which is available to authorized users.

## Introduction

Gold nanoparticles (AuNP) have been widely employed in drug delivery, medical diagnosis, and cancer theranostics as well as consumer products, i.e., cosmetics, food packaging, due to their unique optical and physical properties [1–3]. Upon exposure to a mixture of proteins, NP associate with proteins and form protein corona, which alters the surface chemistry, an adsorbed protein conformations, and the subsequent biological responses, i.e., NP toxicity, cellular NP uptake, catalytic activity of cytochrome P450 (CYP) enzymes toward drugs [4–7]. In vitro studies with primary epithelial cells and cancer cell lines suggest that AuNP was toxic to human hepatocytes, hepatoma cell line C3A, and sperm cells [6–8]. But the formation of protein corona around NP intensively attenuated or potentiated AuNP toxicity in a surface chemistry-dependent manner [6, 7]. Protein corona interfered cellular uptake of AuNP in human hepatocytes, renal proximal tubule cells, HepG2 cells, C3A cell line, irrespective of their sizes and surface charges [6, 7, 9–12].

Hepatic CYP enzymes are primarily involved in the synthesis and/or metabolism of endogenous and exogenous compounds but a wide range of agents, i.e., drugs, pesticides, or NP, reversely affect steroid hormone synthesis, metabolism, and/or detoxification resulting into pharmacological effects and the physiological function [13–17]. Testosterone (TST) is an important androgen and CYP3A4-specific substrate (a main conversion to 6β-OH TST) in a regio- and stereo-selective manner [18]. During phase I metabolism, TST is also hydroxylated to 2β-OH TST by CYP3A4 and dealkylated to androstenedione (AD) by CYP2D6 [17, 19]. In vitro studies with human hepatocyte, C3A cell line, human liver microsomes (HLM), and recombinant CYP enzymes suggested that naked and protein corona-coated AuNP modulated a wide range of CYP enzymes that included CYP1A2, 2C9, 2C19, 2D6, 2E1, and 3A4 [6, 7, 20, 21]. Other metallic NP, naked AgNP also suppressed CYP3A4-mediated production of 6β-OH TST in HLM [22]. AuNP functionalized with branched polyethylenimine (BPEI) and lipoic acid (LA) decreased CYP3A4 activity in C3A cell line but human plasma protein corona (PC) attenuated it [7]. On contrast, naked (no PC) and PC BPEI-AuNP were inhibitory to CYP2C9 and 3A4 in human hepatocytes, irrespective of NP size [6].

In vivo studies reported that small size of AuNP (4 and 13 nm) was accumulated mainly in the liver and spleen in male BALB/c mice and induced expression of hepatic Cyp1a1 and 2b genes [23]. Other metallic NP, zinc oxide NP were inhibitory to hepatic CYP1A2, 2C11, and 3A2 activity in male Sprague Dawley rats with an increase in pathological changes in the liver [24].

To date, little is known how AuNP associate CYP-mediated TST metabolism (hydroxylation and dealkylation of TST) in the absence and/or presence of biologically relevant protein corona. The objectives of this study are to investigate the impact of PC on the physicochemical properties of the 40 and 80 nm cationic BPEI AuNP, anionic LA AuNP, and neutral polyethylene glycol (PEG) AuNP. The impact of AuNP on CYP-mediated TST metabolism with and without PC will be characterized using pHLM. Individual variation in TST metabolism will be also studied within single donor HLM which contained various degree of CYP enzymes.

## Methods/Experimental

### Chemicals

The 2,3,4-^13^C_3_ testosterone (CAS#327048-83-9) and 6β-hydroxytestosterone (6β-OH TST, CAS#62-99-7) were obtained from MilliporeSigma (St. Louis, MO). Testosterone (TST, CAS# 58-22-0), 2α-hydroxytestosterone (2α-OH TST, CAS#004075-14-3), 2β-hydroxytestosterone (2β-OH TST, CAS#10390-14-4), 6α-hydroxytestosterone (6α-OH TST, CAS#2944-87-8), 11β-hydroxytestosterone (11β-OH TST, CAS#1816-85-9), 15β-hydroxytestosterone (15β-OH TST, CAS#39605-73-7), 16α-hydroxytestosterone (16α-OH TST CAS#63-01-4), 16β-hydroxytestosterone (16β-OH TST, CAS#17528-90-4), 11-ketotestosterone (CAS#564-35-2), androstenedione (AD, CAS#63-05-8), 4-hydroxy androstenedione (CAS#566-48-3), and 11β-hydroxy androstenedione (CAS#382-44-5) were purchased from Steraloids (Newport, RI). LC-MS grade-acetonitrile and -formic acid were obtained from Fisher Scientific (Fair Lawn, NJ), while ultrapure water was produced in-house by Synergy® UV-R system from Merck KGaA (Darmstadt, Germany). If not specified, all other reagents were purchased from MilliporeSigma (St. Louis, MO).

### Human Liver Microsomes

Pooled human liver microsomes (pHLM) (200 donors, 100 males, and 100 females) and a single donor liver microsomes were obtained from Corning Inc. (Charlotte, NC). The pHLM are pooled by the supplier but not a pool of a single donor HLM. Characteristics and selected cytochrome P450 (CYP) enzyme activity of the single donor HLM used in this study are presented in Additional file 1: Table S1.

### Gold Nanoparticles Synthesis

Biopure™ 40 and 80 nm spherical AuNP functionalized with cationic branched polyethylenimine (BPEI), anionic lipoic acid (LA), and neutral polyethylene glycol (PEG) were purchased from nanoComposix (San Diego, CA). Core materials were synthesized via the reduction of hydrogen tetrachloroaurate (III) hydrate (HAuCl_4_ 3H_2_O) in potassium carbonate aqueous solution and underwent the aging process and tangential flow filtration (TFF). AuNP surface was functionalized with LA or PEG by adding dihydrolipoic acid (0.2:1, *w*/*w*) or thiol-methoxy-terminated PEG (Laysan Bio Inc., Arab, AL) (0.5:1, *w*/*w*), respectively, with TFF washing and sterile filtration. BPEI-functionalized surfaces were synthesized via EDC/NHS chemistry by linking the carboxylic acid of LA to amines of BPEI. The unbound BPEI was removed with TFF washing and a subsequent centrifugation.

### Preparation of Human Plasma Protein Coronas

Pooled human plasma (HP, *n* = 5) was obtained from Biological Specialty Corp. (Colmar, PA). The 40 and 80 nm AuNP was incubated with human plasma at physiological plasma volume in total blood volume, 55% (*v*/*v*) in an orbital shaking/rotating incubator at 37 °C at 250 rpm for 1 h. At the end of incubation, human plasma protein corona (PC) surrounding NP were collected by centrifugation at 20,000×*g* at 20 °C for 20 min followed by three phosphate-buffered saline (PBS) washes. The unbound and loosely bound proteins were discarded by a centrifugation. The resulting PC AuNP were dispersed in PBS and used for characterization of physicochemical properties and its interaction with TST.

### Physical Characterization of AuNP

Particle size and surface properties were measured by dynamic light scattering (DLS) and transmission electron microscopy (TEM). Hydrodynamic diameters (D_H_), and zeta-potential of the 40 and 80 nm naked (no PC) BPEI-, LA-, and PEG-AuNP in deionized (DI) water and PC AuNP in PBS were measured with the Zetasizer Nano-Zs (Malvern Instruments, Worcestershire, UK) at 0 h at 25 °C. The D_H_, polydispersity index (PDI), and zeta-potential were also obtained for naked and PC AuNP in a microsomal incubation buffer (pH 7.4) at 0 min and 45 min at 37 °C. Samples were measured five times with 11 sub-runs of 10 s each. TEM characterized the morphology of naked and PC AuNP. All AuNP were placed on formvar-coated copper grids and viewed on a Tecnai G2 Spirit BioTWIN with an Oxford detector (FEI Company, Hillsboro, OR) at an accelerating voltage of 120 kV. The GATAN microscopy suite (GATAN Inc., Pleasanton, CA) measured AuNP diameters. An optical absorption spectra was measured with the Spectra Max i3 multi-mode microplate reader (Molecular Devices, Sunnyvale, CA).

### In Vitro Metabolism of Testosterone in the Absence and Presence of Naked and PC AuNP

Preliminary studies were conducted to determine the incubation time and microsomal protein concentrations to provide a linear metabolic rate for TST (a final concentration of 10 μM). The production of TST metabolites was linear from 1.3 to 9.3 mg microsomal protein mL^−1^ for up to 60 min. The metabolic assays were performed as fully described [25]. Briefly, pHLM in a microsomal incubation buffer was treated with 10 μM TST and subsequently, the 40 and 80 nm naked (no PC) AuNP were added at 0, 7, 32, 63, 143, 250, 400, and 571 μg mL^−1^; for PC AuNP pHLM 0, 7, 32, 63, and 143 μg mL^−1^. A microsomal incubation buffer contained 100 mM phosphate buffer, 3.3 mM MgCl_2_, and 1 mM EDTA (pH 7.4). The metabolic reaction was initiated with and without NADPH regenerating system that contained 0.25 mM NADP, 2.5 mM glucose-6-phosphate, and 2 U mL^−1^ glucose-6-phosphate dehydrogenase. After 45-min incubation at 37 °C, the reaction was stopped by adding 4% (*v*/*v*) phosphoric acid aqueous solution (1:1, *v*/*v*). After a centrifuge at 3500 rpm for 20 min, a sample supernatant was collected and stored at − 20 °C until further use. In addition, single donor HLM in a microsomal incubation buffer was treated with 10 μM TST followed by incubation with the 63 μg mL^−1^ of all naked and PC AuNP for 45 min at 37 °C. At the end of incubation, the sample was processed and stored at − 20 °C as above.

### Standards and Sample Preparation

Primary standard stock solutions of TST, its metabolites, and ^13^C_3_-labeled TST as an internal standard (ISTD) were prepared in methanol at a concentration of 1 mM and stored at − 20 °C until further use. The concentrations for working standard solutions of TST and its metabolites were 0.01, 0.05, 0.1, 0.5, 1, 5, 10, 50, 100, and 200 μM with serial dilutions of primary stock solution. For standard calibrators, a 50 μL aliquot of each working standard solution was added to 450 μL of reaction buffer, resulting in 1:10 dilutions, while 0.1 μM of ISTD solution was also prepared using 4% phosphoric acid water solution. Quality control (QC) samples were prepared at concentrations of 0.01, 0.05, and 0.1 μM.

After thawing, samples underwent centrifugation at 3500 rpm for 20 min at room temperature. Supernatant was spiked with 50 μL of 0.1 μM of ISTD and underwent Oasis PRIME HLB 96-well μelution plate and a collection plate in the Waters positive pressure-96 processor at 80 psi for 1–2 min. (Waters Corp., Milford, MA). After washing with 300 μL of water with 5% methanol and elution with 50 μL of acetonitrile/methanol mixture (90/10, *v*/*v*), the resulting eluent was diluted in 50 μL water (a final volume of 100 μL) and subject to liquid chromatography-mass spectrometry (LC-MS/MS).

### Liquid Chromatography-Mass Spectrometry

All samples were separated on Waters UPLC HSS T3 column (2.1 × 50 mm, 1.8 μm) with Waters Acquity Ultra Performance Liquid Chromatography system with Triple Quadrupole Detector (UPLC TQD) (Waters Corp., Milford, MA). The mobile phases A and B were 0.1% formic acid in water and 0.1% formic acid in methanol, respectively. A gradient LC method was used at a flow rate of 600 μL min^−1^ for 0–8.4 min. The gradient was 0–1 min (30% B), 1–3 min (to 50% B), 3–3.5 min (50% B), 3.5–7 min (to 80% B), 7–7.01 min (to 98% B), 7.01–7.5 min (98% B), and 7.51–8.4 min (30% B). The MS conditions were described briefly below. The ionization source was operated in electrospray positive (ESI^+^) mode with capillary voltage 4000 V; for source temperature 150 °C; and for desolvation temperatures 450 °C. The flow rates of desolvation gas (N_2_), cone gas (N_2_), and collision gas (argon) were 900 L h^−1^, 100 L h^−1^, and 0.1 mL min^−1^, respectively. The scan type was multiple reaction monitoring (MRM) and the MS run time was 8.4 min. The MRM transitions used for the analysis were summarized in Additional file 1: Table S2 and Fig. 2. The injection volume was 2 μL and the column was maintained at 50 °C throughout the analysis. All quantitation methods were based on a seven-point calibration curve over concentration ranges from 0.001 to 20 μM. The limit of detection (LOD) and limit of quantitation (LOQ) of 0.001 μM and 0.005 μM were established for TST and targeted metabolites.

### Statistical Analysis

The effects of dispersants on D_H_ and PDI of naked and PC on AuNP were assessed using the Student’s *t* test with a two-tailed distribution. Half maximal inhibitory concentration (IC_50_) and half maximal activation concentration (EC_50_) of AuNP toward the production of CYP-dependent TST metabolites in pHLM were determined by fitting a Hill equation with variable slope to the observed data using GraphPad Prism®. A one-way analysis of variance (ANOVA) was conducted using GraphPad Prism® to assess the effects of AuNP treatment on TST metabolism in single donor HLM. When effects were significant, a multiple comparison was performed with Tukey’s honest significant difference (HSD) test at 5% level of significance. Pearson correlation coefficient (*r*) between CYP activity of single donor HLM and the production of CYP-dependent TST metabolites was determined using GraphPad Prism® version 6.07 (La Jolla, CA).

## Results and Discussion

### Physicochemical Characterization of Naked and Human Plasma Protein Corona AuNP

**Fig. 1:**
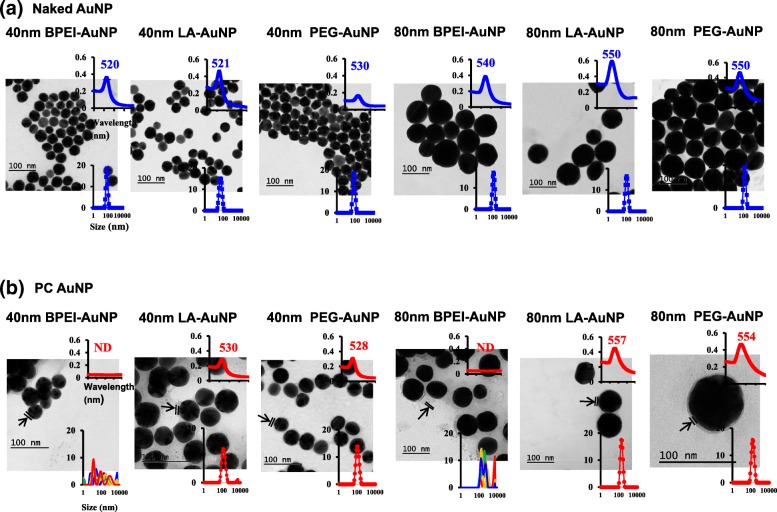
Transmission electron micrographs of (**a**) the 40 and 80 nm naked AuNP in deionized water and **b** the 40 and 80 nm PC AuNP in PBS at 0 h at 25 °C, UV absorption spectrum (upper inset), and the dynamic light scattering distribution (lower inset). Arrows indicate PC formation. *BPEI* branched polyethylenimine, *LA* lipoic acid, *PEG* polyethylene glycol, *ND* not determined, *PC* human plasma protein corona, *naked* no PC

The impact of human plasma protein corona (PC) in NP size, surface charge, and morphology as well as a spectral property has been characterized using DLS, TEM, and UV-Vis spectroscopy (Fig. 1). TEM images demonstrated that all naked (no PC) and PC AuNP except for 40 and 80 nm PC BPEI-AuNP were monodisperse with the steady size distribution and unique UV-Vis spectrum ranges (520–557 nm) (Fig. 1a, b). Distinct PC around AuNP in PBS were also found by TEM. Aggregation of the 40 and 80 nm PC coated branched polyethylenimine (BPEI)-AuNP in PBS at 0 min at 25 °C correlated multiple peaks in size distribution and redshifts of the absorption spectra relative to naked BPEI-AuNP (Fig. 1b). The hydrodynamic diameter (D_H_) values of the 40 and 80 nm PC BPEI-AuNP dissolved in PBS at 0 min at 25 °C and in microsomal incubation buffer at 0 and 45 min at 37 °C were not determined by DLS, along with multiple peaks in size distribution. The D_H_ values of the 40 nm naked BPEI- and LA-AuNP and PC PEG-AuNP and the 80 nm naked BPEI-AuNP in microsomal incubation buffer substantially increased up to 45 min at 37 °C, whereas its value decreased for the 80 nm PC LA-AuNP (Table 1). The polydispersity index (PDI) of the 40 nm naked and PC PEG-AuNP and 80 nm PC PEG-AuNP increased at 45 min at 37 °C. In addition, zeta (z) potential values of the 40 and 80 nm naked BPEI-AuNP and 40 nm naked PEG-AuNP substantially decreased over time. Previous study reported that AuNP (7 and 70 nm) associated with human liver microsomal proteins altered the characteristic absorbance maximum in UV-visible range [21]. These results were supported by the recent studies in our laboratory that PC and human serum albumin corona altered NP size, red-shift of maximum absorbance, and morphology irrespective of a dissolving medium and an incubation time [6, 7, 10, 26]. Based on changes in PC-mediated NP physicochemical properties and enzymatic function of CYP activities [6, 7], the potential effect of the 40 and 80 nm AuNP on CYP-mediated human liver microsomal TST metabolism was investigated in the presence of a more biologically relevant PC.

**Table 1:** Hydrodynamic diameters and polydispersity index (PDI) and zeta (z)-potential of the 40 and 80 nm bare and PC AuNP with cationic BPEI, anionic LA, and neutral PEG in a microsomal incubation buffer (100 mM phosphate buffer) at 0 min and 45 min at 37 °C. Data is mean ± S.D. (*n* = 5)

AuNP size (nm)	Surface coatings	Hydrodynamic diameters (nm)		PDI		z-potential	
		0 min	45 min	0 min	45 min	0 min	45 min
40	BPEI	498.5 ± 25.4	807.6 ± 51.8***	0.38 ± 0.05	0.40 ± 0.04	− 8.2 ± 2.6	−18.0 ± 1.1**
	LA	584.5 ± 12.7	816.4 ± 0.5**	0.36 ± 0.04	0.38 ± 0.02	−22.6 ± 1.8	−21.6 ± 0.9
	PEG	685.4 ± 13.5	715.2 ± 41.9	0.32 ± 0.02	0.43 ± 0.02**	− 7.6 ± 0.5	− 9.1 ± 0.6**
	PC LA	165.5 ± 0.1	168.1 ± 0.1	0.48 ± 0.07	0.50 ± 0.13	−11.5 ± 1.1	− 11.8 ± 1.7
	PC PEG	73.5 ± 0.02	77.3 ± 1.3**	0.09 ± 0.02	0.18 ± 0.01***	−9.5 ± 1.5	− 11.8 ± 1.1
80	BPEI	764.4 ± 14.6	925.9 ± 58.2**	0.40 ± 0.06	0.42 ± 0.04	−6.3 ± 0.6	−12.7 ± 0.8***
	LA	835.0 ± 46.5	859.1 ± 63.1	0.45 ± 0.02	0.51 ± 0.06	−29.9 ± 1.8	− 29.5 ± 1.3
	PEG	113.0 ± 0.60	111.4 ± 0.6	0.09 ± 0.01	0.11 ± 0.01	−11.0 ± 1.3	−12.3 ± 1.2
	PC LA	133.2 ± 0.01	128.1 ± 0.02**	0.10 ± 0.01	0.09 ± 0.02	−12.0 ± 1.1	−12.3 ± 0.8
	PC PEG	126.5 ± 0.01	128.4 ± 1.6	0.08 ± 0.01	0.12 ± 0.01**	−8.7 ± 4.4	−11.2 ± 1.9

*BPEI* branched polyethylenimine, *LA* lipoic acid, *PEG* polyethylene glycol, *PC* human plasma protein corona

*p < 0.05; **p < 0.005; ***p < 0.0001

### AuNP-Mediated Testosterone Metabolism in Pooled Human Liver Microsomes

**Fig. 2:**
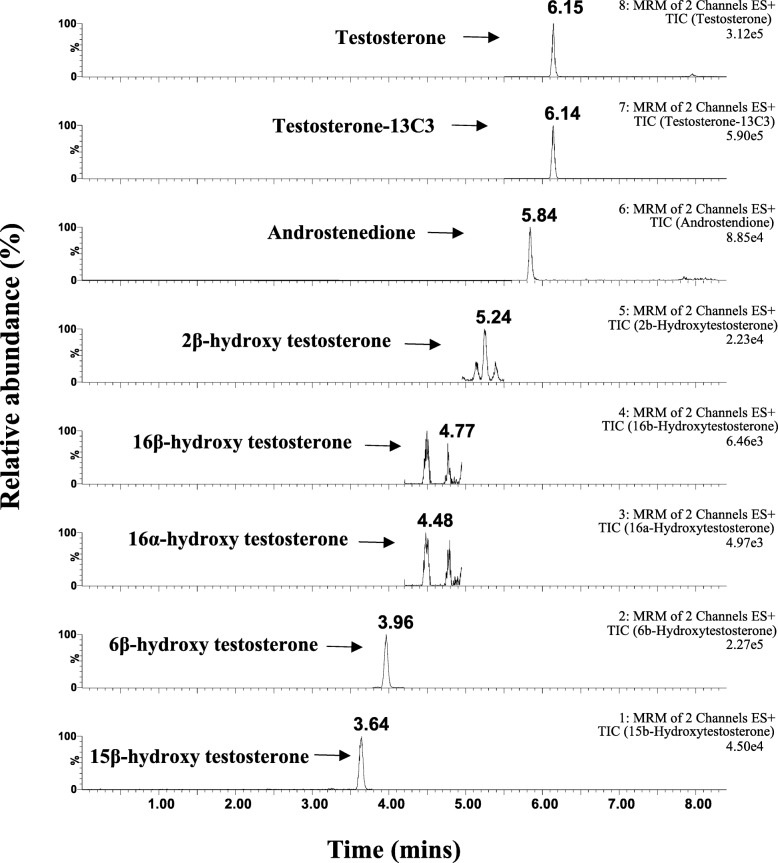
The extracted ion chromatogram (XIC) for testosterone (TST), ^13^C3-labeled TST, androstenedione, 2β-hydroxy testosterone (2β-OH TST), 16α-hydroxytestosterone (16α-OH TST), 16β-hydroxytestosterone (16β-OH TST), 6β-hydroxytestosterone (6β-OH TST), and 15β-hydroxytestosterone (15β-OH TST) produced in pooled HLM at the final concentration of 10 μM testosterone in the presence of NADPH for 45 min at 37 °C. *HLM* human liver microsomes, *NADPH* a reduced nicotinamide adenine dinucleotide phosphate

A total of 11 metabolites of TST were screened and six metabolites were found in pooled human liver microsomes (pHLM) at 10 μM TST. The targeted LC-MS/MS analysis of TST and the six selected metabolites is demonstrated in Fig. 2. The list of the selected metabolites included five hydroxylated TST metabolites (2β-OH TST, 6β-OH TST, 15β-OH TST, 16α-OH TST, 16β-OH TST) and a dealkylated metabolite (androstenedione, AD). This correlates with the previous studies using human hepatocytes and HLM that TST was mainly hydroxylated into 6β-OH TST and to a lesser degree 2β-OH TST, 15β-OH TST, 16α-OH TST, and 16β-OH TST as well as a dealkylated metabolite, AD [17, 19, 27].

**Fig. 3:**
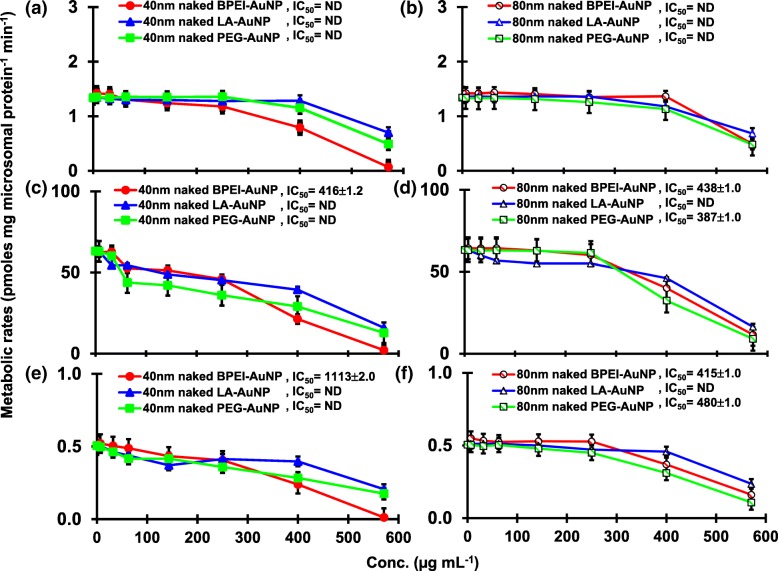
Inhibitory effect of naked (no PC) AuNP on the production of 2β-OH TST (**a**, **b**), 6β-OH TST (**c**, **d**) and 15β-OH TST (**e**, **f**) in pHLM. Data represent mean ± S.D. (*n*=3). *IC*_50_, half maximal inhibitory conc.; *pHLM*, pooled human liver microsomes; *ND*, not determined by fitting a Hill equation with variable slope to the observed data using GraphPad Prism®; *PC*, human plasma protein; *BPEI*, branched polyethylenimine; *LA*, lipoic acid; PEG, polyethylene glycol; Conc, concentration; *2β-OH TST, 2β*-hydroxytestosterone; *6β-OH TST, 6β*-hydroxytestosterone; *15β-OH TST, 15β*-hydroxytestosterone

**Fig. 4:**
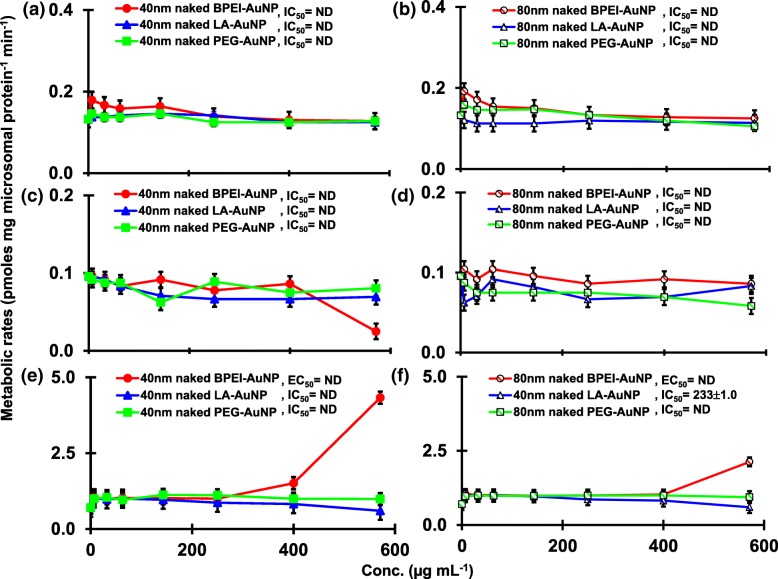
An inhibitory and stimulatory effect of naked (no PC) AuNP on the production of 16β-OH TST (**a**, **b**), 16β-OH TST (**c**, **d**) and AD (**e**, **f**) in pHLM. Data represent mean ± S.D. (*n*=3). *IC*_50_, half maximal inhibitory conc.; *EC*_50_, half maximal activation conc.; *pHLM*, pooled human liver microsomes; *ND*, not determined by fitting a Hill equation with variable slope to the observed data using GraphPad Prism®; *PC*, human plasma protein; *BPEI*, branched polyethylenimine; *LA*, lipoic acid; *PEG*, polyethylene glycol; Conc, concentration; *16α-OH TST, 16α*-hydroxytestosterone; *16β-OH TST, 16β*-hydroxytestosterone; *AD*, androstenedione.

**Fig. 5:**
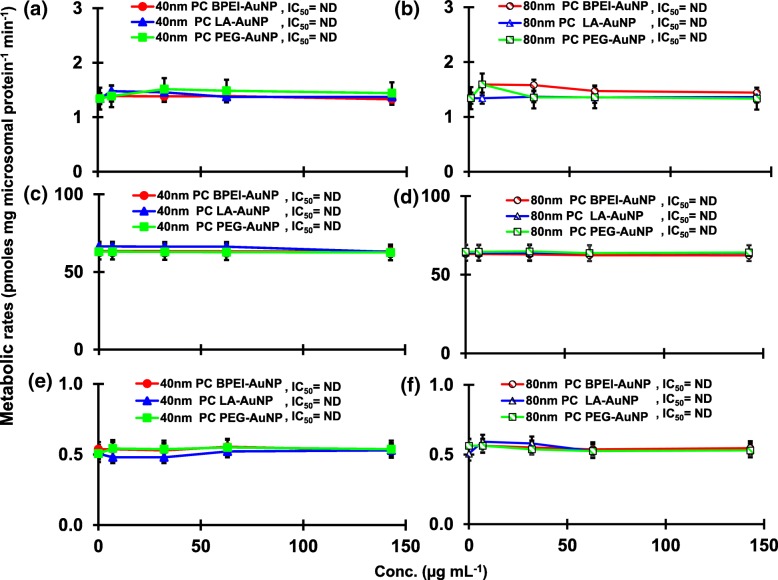
Effects of PC AuNP on the production of 2β-OH TST (**a**, **b**), 6β-OH TST (**c**, **d**) and 15β-OH TST (**e**, **f**) in pHLM. Data represent mean ± S.D. (*n*=3). *IC*_50_, half maximal inhibitory conc.; *pHLM*, pooled human liver microsomes; *ND*, not determined by fitting a Hill equation with variable slope to the observed data using GraphPad Prism®; *PC*, human plasma protein corona; *BPEI*, branched polyethylenimine; *LA*, lipoic acid; *PEG*, polyethylene glycol; Conc, concentration; *2β-OH TST, 2β*-hydroxytestosterone; *6β-OH TST, 6β*-hydroxytestosterone; *15β-OH TST,* 15β-hydroxytestosterone.

**Fig. 6:**
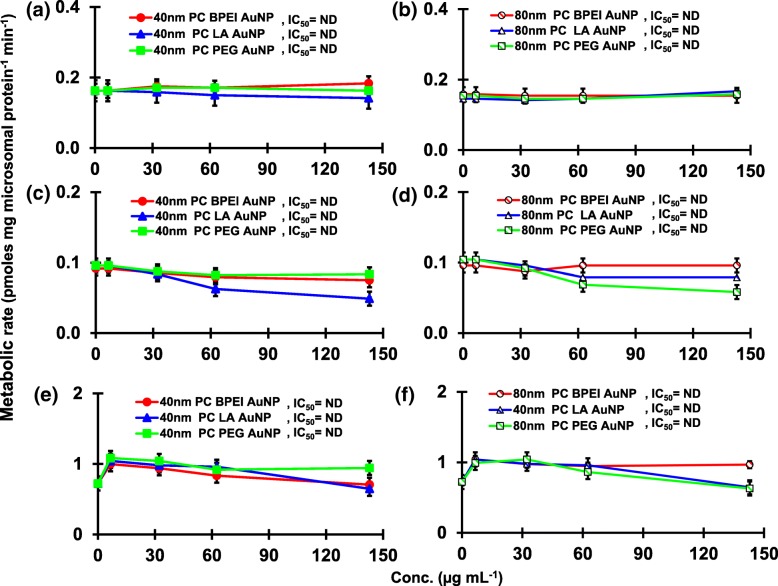
Effects of PC AuNP on the production of 16α-OH TST (**a**, **b**), 16β-OH TST (**c**, **d**) and AD (**e**, **f**) in pHLM. Data represent mean ± S.D. (*n*=3). *IC*_50_, half maximal inhibitory conc.; *pHLM*, pooled human liver microsomes; *ND*, not determined by fitting a Hill equation with variable slope to the observed data using GraphPad Prism®; *PC*, human plasma protein corona; *BPEI*, branched polyethylenimine; *LA*, lipoic acid; *PEG*, polyethylene glycol; Conc, concentration; *16α-OH TST, 16α*-hydroxytestosterone; *16β-OH TST, 16β*-hydroxytestosterone; *AD*, androstenedione.

**Fig. 7:**
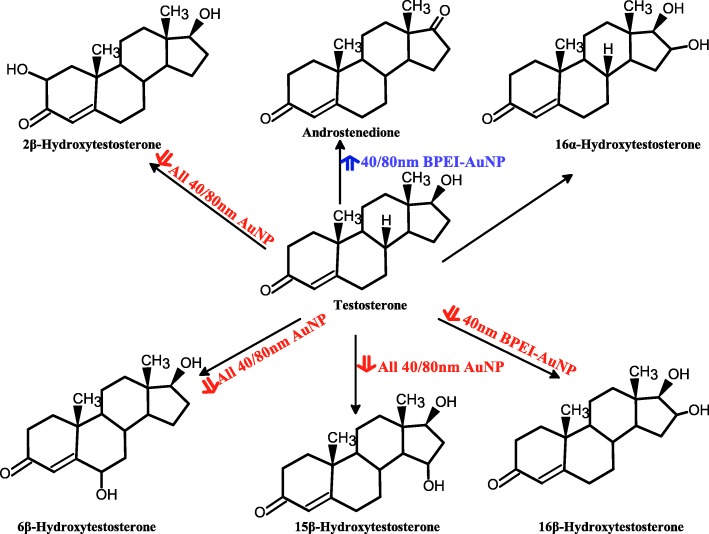
Proposed scheme of testosterone metabolism in pooled human liver microsomes and AuNP-mediated its metabolites production. *AuNP* gold nanoparticles, *BPEI* branched polyethylenimine, *LA* lipoic acid, *PEG* polyethylene glycol, *PC* human plasma protein corona. Red arrow, inhibitory effect; blue arrow, stimulatory effect

The result of coincubation of TST with the 40 and 80 nm naked AuNP in pHLM was shown in Figs. 3 and 4. All 40 and 80 nm naked AuNP altered the production of 2β-OH TST, 6β-OH TST, and 15β-OH TST in pHLM with varying degree of inhibition (Fig. 3a–f). Half maximal inhibitory concentration (IC_50_) values of the 40 nm BPEI-AuNP for the production of 6β-OH TST was 416 μg mL^−1^; for 80 nm BPEI-AuNP 438 μg mL^−1^; and for 80 nm PEG-AuNP 387 μg mL^−1^ (Fig. 3c, d). For 15β-OH TST production, IC_50_ values of the 40 nm BPEI-AuNP was 1113 μg mL^−1^; for 80 nm BPEI-AuNP 415 μg mL^−1^; and for 80 nm PEG-AuNP 480 μg mL^−1^ (Fig. 3e, f). These results were supported by in vitro studies with human cancer cell lines and liver tissues that a metallic NP, AgNP, and porous silicon NP impeded 6β-OH TST production in human epithelial colorectal adenocarcinoma Caco2 cells, hepatocellular carcinoma HepG2 cells, and human liver microsomes [22, 28]. The 40 and 80 nm naked AuNP were not inhibitory to the production of 16α-OH TST and 16β-OH TST except for the 40 nm BPEI-AuNP which suppressed 16β-OH TST production at the highest concentration (517 μg mL^−1^) (Fig. 4a–d). The 40 and 80 nm BPEI-AuNP increased the production of androstenedione (AD) at the highest concentration with the corresponding metabolic rates of 4.3 and 2.1 pmol mg protein^−1^ min^−1^, respectively compared to that in control (0.7 pmol mg protein^−1^ min^−1^) (Fig. 4e, f). But the 80 nm LA-AuNP was inhibitory to AD production with IC_50_ value of 233 μg mL^−1^. These results indicated that the naked AuNP mediated the production of the selected TST metabolite in the surface-coating, and size-dependent manners. In addition, all 40 and 80 nm PC AuNP were not inhibitory to the production of six selected metabolites from TST in pHLM at up to 143 μg mL^−1^, irrespective of surface coatings (Figs. 5 and 6). Especially, PC alleviated the 40 nm naked BPEI-AuNP-mediated inhibition of 6β-OH TST and 15β-OH TST production at higher concentrations (32 μg mL^−1^ to 143 μg mL^−1^) (Figs. 3c, e and 5c, e). This results exhibited that the 40 and 80 nm naked BPEI-, LA-, and PEG-AuNP decreased TST hydroxylation (2β-OH TST, 6β-OH TST, and 15β-OH TST) in a dose-dependent manner (Fig. 7). In addition, the 40 and 80 nm naked BPEI-AuNP increased AD production but the former decreased 16β-OH TST. In vitro study reported that 6β-OH TST production mainly mediated by CYP3A4 was inhibited by single-walled carbon nanotubes (SWCNT) in a dose-dependent manner but bovine serum albumin corona alleviated it [17, 29]. Our laboratory recently reported that the 40 and 80 nm naked and PC BPEI-AuNP served as an inhibitor for CYP1A2, 2C9, and 3A4 at the cellular and transcriptional levels [6, 7]. In vivo study reported that PEG-AuNP (4 and 13 nm) was mainly accumulated mainly in the liver in male BALB/c mice and altered transcriptional levels of hepatic Cyp1a1 and 2b genes [23]. Male ICR mice with i.v. injection of PEG-NH_2_-AuNP exhibited NP increased plasma TST level without the sperm morphology and fertility [30]. Other metallic NP, titanium dioxide (TiO_2_) was accumulated in testis of CD1 male mice and decreased cyp1b1 and 2e1 expression [31]. Epidemiology study reported that adult male at Massachusetts infertility clinic showed low plasma TST levels collated with a high level of 3,5,6-trichloro-2-pyridinol(TCP) derived from high exposure of chlorpyrifos (CFS), a known endocrine disruptor and an inhibitor for CYP-mediated TST metabolism [32]. Previous study reported that the known endocrine-disrupting insecticides, CFS, CFS oxon, fonofos, phorate, diethyltoluamide (DEET), and permethrin substantially inhibited and/or activated the production of the hydroxylated and/or dealkylated TST metabolites, i.e., 2β-OH TST, 6β-OH TST, 15β-OH TST, and AD and 4-hydroxy AD in human liver [17]. With this said, it is reasonable to postulate that AuNP may be a potential endocrine disruptor by mediating an inhibitory and/or activating potencies against CYP-mediated metabolism of TST.

### TST Metabolism in Single Donor Human Liver Microsomes and Its Modulation by AuNP

The production of six selected metabolites were characterized with single donor HLM isolated from donors with varying degree of CYP activity at the pHLM-based non-inhibitory concentration of the 40 and 80 nm naked and PC AuNP (10 μg mL^−1^). An individual variation in TST metabolism was observed among three different single donor HLM (Additional file 1: Figure S1). The relationship between the catalytic activity of each CYP enzymes and the production of TST-derived metabolites was characterized within three different single donor HLM that contained a low, a medium, and a high CYP catalytic activity (Additional file 1: Table S1). The production of 6β-OH TST positively correlated with the activity of CYP2C19 (*r* = 0.99 and *p* = 0.01) and CYP3A4 (*r* = 0.99 and *p* = 0.03) within individuals (Additional file 1: Figure S2). AD production negatively correlated with CYP4A11 (*r* = − 0.98 and *p* = 0.04) (Additional file 1: Figure S3). These results were consistent with a previous study reported that CYP3A4 and CYP2D6 played a key role in the production of a major TST metabolite, 6β-OH TST and AD, respectively [17]. This study also suggested that an individual variation in CYP-dependent metabolism of TST depends on the genotypes of CYP enzymes and their phenotypic activities. A previous study reported that CYP polymorphisms and phenotypes are the key features in CYP function and result in a categorical pharmacogenetics phenotypes as poor, intermediate, extensive, and ultrarapid metabolizers contributing to an individual susceptibility to adverse drug reactions and/or drug efficacy and dose suggesting that poor metabolizer of CYP enzyme, i.e., CYP3A4 may be susceptible to TST metabolism from exposure to CYP inhibitor, AuNP [33].

**Fig. 8:**
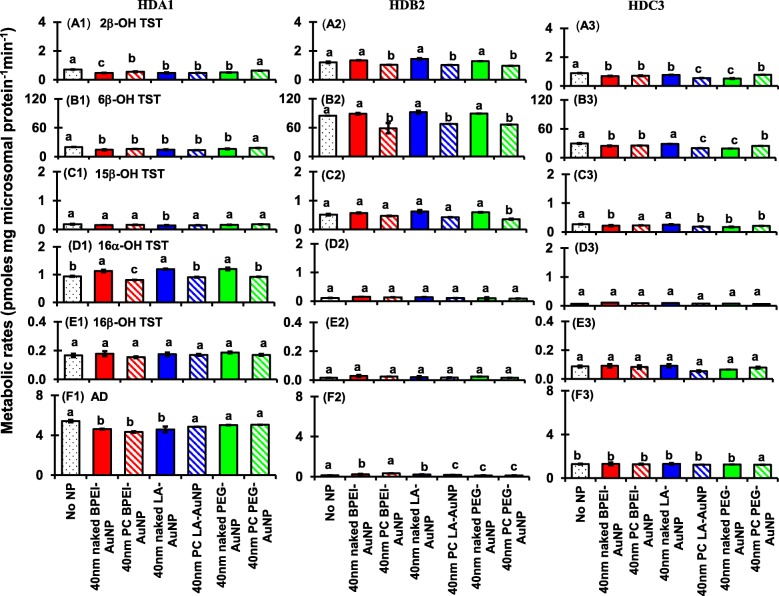
Effects of 40 nm naked and PC AuNP on the production of 2β-OH TST (A1–A3), 6β-OH TST (B1–B3), 15β-OH TST (C1–C3), 16α-OH TST (D1–D3), 16β-OH TST (E1–E3), and AD (F1–F3); in three different single donor HLM (HDA1, HDB2, and HDC3). Means followed by the same letter were not significantly different for each nanoparticle (Tukey’s honest significant difference = 5%). *Naked* no PC, *PC* human plasma protein corona, *HLM* human liver microsomes, *BPEI* branched polyethylenimine, *LA* lipoic acid, *PEG* polyethylene glycol, *2β*-*OH TST* 2β-hydroxytestosterone, *6β*-*OH TST* 6β-hydroxytestosterone, *15β*-*OH TST* 15β-hydroxytestosterone, *16α*-*OH TST* 16α-hydroxytestosterone, *16β*-*OH TST* 16β-hydroxytestosterone, *AD* androstenedione

**Fig. 9:**
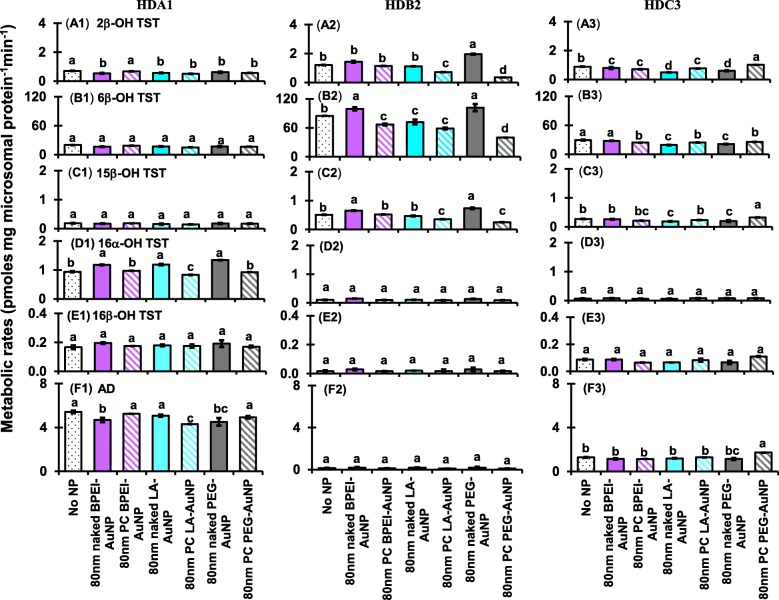
Effects of 80 nm naked and PC AuNP on the production of 2β-OH TST (A1–A3), 6β-OH TST (B1–B3), 15β-OH TST (C1–C3), 16α-OH TST (D1–D3), 16β-OH TST (E1–E3), and AD (F1–F3); in three different single donor HLM (HDA1, HDB2, and HDC3). Means followed by the same letter were not significantly different for each nanoparticle (Tukey’s honest significant difference = 5%). *Naked* no PC, *PC* human plasma protein corona, *HLM* human liver microsomes, *BPEI* branched polyethylenimine, *LA* lipoic acid, *PEG* polyethylene glycol, *2β*-*OH TST* 2β-hydroxytestosterone, *6β*-*OH TST* 6β-hydroxytestosterone, *15β*-*OH TST* 15β-hydroxytestosterone, *16α*-*OH TST* 16α-hydroxytestosterone, *16β*-*OH TST* 16β-hydroxytestosterone, *AD* androstenedione

As shown in Figs. 8 and 9, coincubation of TST with AuNP at non-inhibitory concentration caused an increase and/or decrease in CYP-mediated metabolism of TST among single donor HLM as function of size and surface change modification. ANOVA indicated that significant changes by AuNP size (*p* < 0.0001), surface coatings (*p* < 0.0001), and PC formation (*p* < 0.0001) were observed for the production of six selected metabolites of TST in single donor HLM (HDA1, HDB2, and HDC3).

All 40 nm naked and PC AuNP decreased both 2β-OH TST and 6β-OH TST production in HDA1 and HDC3 except for PC PEG-AuNP in the former and naked LC-AuNP in the latter, whereas in HDB2, only PC AuNP potentiated inhibition of their productions, irrespective of surface coatings (Fig. 8(A1–B3)). The 40 nm naked LA-AuNP was an inhibitor for 15β-OH TST production in HDA1; for HDB2 PC PEG-AuNP; and for HDC3 naked BPEI- and PEG-AuNP and PC LA- and PC PEG-AuNP (Fig. 8(C1–C3)). All 40 nm naked AuNP were an activator for 16α-OH TST production in HDA1 but PC attenuated it except for PC BPEI-AuNP which potentiated its inhibition (Fig. 8(D1)). All 40 nm naked and PC AuNP did not influence the production of 16β-OH production within individuals (Fig. 8(E1–E3)). AD production was modulated by the 40 nm naked and PC AuNP with varying degrees of inhibition except for PC PEG-AuNP which served an activator in HDC3 (Fig. 8(F1–F3)).

The 80 nm naked and PC AuNP-mediated inhibition for 2β-OH TST production was observed within individuals except for 80 nm naked and PC PEG-AuNP which served as the activators in HDB2 and HDC3, respectively (Fig. 9(A1–A3)). These results were not consistent with all naked and PC 40 nm AuNP-mediated inhibition for 2β-OH TST, irrespective of surface coatings (Fig. 8(A1–A3)). For 6β-OH TST, the 80 nm naked AuNP were the activators except for LA-AuNP but PC potentiated its inhibition in HDB2 (Fig. 9(B2)), which was similar to the 40 nm naked and PC AuNP-mediated inhibition and/or activation, respectively (Fig. 8(B2)). For 15β-OH TST, 80 nm naked BPEI- and PEG-AuNP and PC PEG-AuNP were the activators in HDB2 and in HDC3, respectively (Fig. 9(C2 and C3)). The 80 nm naked AuNP increased 16α-OH TST production in HDA1, irrespective of surface coatings but PC attenuated it except for PC LA-AuNP which was an inhibitor (Fig. 9(D1)). This is similar to the 40 nm naked and PC AuNP-mediated activation and attenuation for 16α-OH TST production in HDA1 (Fig. 8(D1)). All 80 nm naked and PC were not inhibitory to 16β-OH TST production within all individuals, irrespective of surface coatings (Fig. 9(E1–E3)). These results were consistent with the 40 nm naked and PC-mediated its production within individuals (Fig. 8(E1–E3)). For AD production, the 80 nm naked BPEI- and PEG-AuNP were the inhibitors but PC attenuated and vice versa with naked and PC LA-AuNP in HDA1 (Fig. 9(F1)). The 80 nm naked and PC AuNP decreased its production in HDC3 except for PC PEG-AuNP, which was an activator (Fig. 9(F3)). This study strongly suggests that AuNP interaction with CYP enzymes in HLM cause a decrease and/or increase in TST conversion to hydroxylated and dealkylated metabolites within individuals and the presence of PC played the inhibitive or protective role. In vivo study reported that the male CD-1 mice orally administrated with ketoconazole, a noncompetitive CYP3A4 inhibitor showed that a decrease in serum TST level, gonadal TST secretion, and hepatic TST hydroxylation activity that included 6β-OH TST, 15α-OH TST, 15β-OH TST, and 16β-OH TST [34]. In vitro studies with human hepatocyte, C3A cell line, HepG2 cell line, HLM, and recombinant CYP enzymes suggested that AuNP modulated the activity of various CYP enzymes that included CYP1A2, 2C9, 2C19, 2D6, 2E1, and 3A4 [6, 7, 20, 21]. PC and human serum albumin corona mitigated an inhibitory effect of BPEI- and LA-AuNP on CYP1A2, 2C9, and 3A4 enzyme activity in human hepatocytes and C3A cell line [6, 7]. That being said, it may be rational to propose that AuNP interference with CYP enzymes relates individual susceptibility to unexpected toxicological effects that may result in an altered circulating TST level tied to endocrine disrupting substance and/or drug-drug interaction sharing the same CYP enzymes [35].

## Conclusions

These studies exhibit that AuNP interaction with PC definitely modulate CYP-dependent metabolism of TST in HLM derived from a large donor pool that better represents the average American population. The 40 nm naked (no PC) AuNP and to a lesser degree 80 nm naked AuNP inhibited TST hydroxylation but activated TST dealkylation at high concentration. Cationic BPEI-AuNP withheld the production of 6β-OH TST and 15β-OH TST in pooled HLM but the presence of a more biologically relevant PC alleviated their adverse effects as function of size and surface charge modification. In most cases, the 40 and 80 nm naked and PC AuNP are essentially inhibitory to TST metabolism in single donor HLM in a surface chemistry-dependent manner at the noninhibitory concentration. In addition, PC PEG-AuNP caused an activation of AD production in HDC3, irrespective of size. These results may indicate that individual variations in AuNP-mediated TST metabolism could be a factor for their toxicity and could be utilized to identify vulnerable subgroup to TST-disrupting NP.

## Additional file

Additional file 1: **Figure S1.** Production of testosterone metabolites within three different single donor HLM (HDA1, HDB2 and HDC3). Data represent mean ± S.D. (*n* = 3). 2β-OH TST, 2β-hydroxytestosterone; 6β-OH TST, 6β-hydroxytestosterone; 15β-OH TST, 15β-hydroxytestosterone; 16α-OH TST, 16α-hydroxytestosterone; 16β-OH TST, 16β-hydroxytestosterone; AD, androstenedione. **Figure S2.** Pearson correlation coefficient (r) and p-value (p) between CYP activity and the production of TST-derived metabolites in single donor HLM. The catalytic activity of seven CYP enzymes of single donor HLM was adapted from Corning **Table S2.** CYP, cytochrome P450; 2β-OH TST, 2β-hydroxytestosterone; 6β-OH TST, 6β-hydroxytestosterone; 15β-OH TST, 15β-hydroxytestosterone; HLM, human liver microsomes. **Figure S3.** Pearson correlation coefficient (r) and p-value (p) between CYP activity and the production of TST-derived metabolites in single donor HLM. The catalytic activity of seven CYP enzymes of single donor HLM was adapted from Corning **Table S2.** CYP, cytochrome P450; TST, testosterone; 16α-OH TST, 16α-hydroxytestosterone; 16β-OH TST, 16β-hydroxytestosterone; AD, androstenedione; HLM, human liver microsomes.**Table S1.** Characterization and selected CYP enzyme activity of single donor human liver microsomes. **Table S2.** MRM parameters for determination of testosterone, metabolites and 13C3-testosterone (ISTD) (PDF 898 kb)

## Data Availability

All data generated or analyzed during this study are included in this article and its supplementary information file.

## Abbreviations

11β-OH TST: 11β-hydroxytestosterone
15β-OH TST1: 5β-hydroxytestosterone
16α-OH TST: 16α-hydroxytestosterone
16β-OH TST: 16β-hydroxytestosterone.
2α-OH TST: 2α-hydroxytestosterone
2β-OH TST: 2β-hydroxytestosterone
6α-OH TST: 6α-hydroxytestosterone
6β-OH TST: 6β-hydroxytestosterone
AD: Androstenedione
AgNP: Silver nanoparticles
ANOVA: One-way analysis of variance
AuNP: Gold nanoparticles
BPEI: Branched polyethylenimine
CFS: Chlorpyrifos
CYP: Cytochrome P450
DEET: Diethyltoluamide
D_H_: Hydrodynamic diameters
DI: Deionized water
DLS: Dynamic light scattering
EC_50_: Half maximal activation concentration
EDC/NHS: 1-Ethyl-3-(3-dimethylaminopropyl) carbodiimide/N-hydroxysuccinimide
ESI^+^: Electrospray positive
HLM: Human liver microsomes
HSD: Tukey’s honest significant difference
IC_50_: Half maximal inhibitory concentration
ISTD: Internal standard
LA: Lipoic acid
LC-MS/MS: Liquid chromatography-mass spectrometry
LOD: Limit of detection
LOQ: Limit of quantitation
MRM: Multiple reaction monitoring
NADP: Nicotinamide adenine dinucleotide phosphate
NADPH: Reduced NADP
naked: No PC
NP: Nanoparticles
PBS: Phosphate-buffered saline
PC: Human plasma protein corona
PDI: Polydispersity index
PEG: Polyethylene glycol
pHLM: Pooled human liver microsomes
QC: Quality control
SWCNT: Single-walled carbon nanotube
TEM: Transmission electron microscopy
TFF: Tangential flow filtration
TiO_2_: Titanium dioxide
TST: Testosterone
UPLC TQD: Ultra performance liquid chromatography system with Triple quadrupole Detector
