# Supplementary material for: Impact of Gold Nanoparticles on Testosterone Metabolism in Human Liver Microsomes
Source: Nanoscale Res Lett. 2019 Jun 17;14:205. doi: 10.1186/s11671-019-3021-z (PMC6579798; doi:10.1186/s11671-019-3021-z)
Supplement: Supplementary file 1 — Figure S1. Production of testosterone metabolites within three different single donor HLM (HDA1, HDB2 and HDC3). Data represent mean ± S.D. (n = 3). 2β-OH TST, 2β-hydroxytestosterone; 6β-OH TST, 6β-hydroxytestosterone; 15β-OH TST, 15β-hydroxytestosterone; 16α-OH TST, 16α-hydroxytestosterone; 16β-OH TST, 16β-hydroxytestosterone; AD, androstenedione. Figure S2. Pearson correlation coefficient (r) and p-value (p) between CYP activity and the production of TST-derived metabolites in single donor HLM. The catalytic activity of seven CYP enzymes of single donor HLM was adapted from Corning Table S2. CYP, cytochrome P450; 2β-OH TST, 2β-hydroxytestosterone; 6β-OH TST, 6β-hydroxytestosterone; 15β-OH TST, 15β-hydroxytestosterone; HLM, human liver microsomes. Figure S3. Pearson correlation coefficient (r) and p-value (p) between CYP activity and the production of TST-derived metabolites in single donor HLM. The catalytic activity of seven CYP enzymes of single donor HLM was adapted from Corning Table S2. CYP, cytochrome P450; TST, testosterone; 16α-OH TST, 16α-hydroxytestosterone; 16β-OH TST, 16β-hydroxytestosterone; AD, androstenedione; HLM, human liver microsomes.Table S1. Characterization and selected CYP enzyme activity of single donor human liver microsomes. Table S2. MRM parameters for determination of testosterone, metabolites and 13C3-testosterone (ISTD) (PDF 898 kb) [file 11671_2019_3021_MOESM1_ESM.pdf]

Supplementary materials:

Impact of Gold Nanoparticles on Testosterone Metabolism in Human Liver Microsomes

Kyoungju Choi* and Hyun Joo

Department of Anatomy & Physiology, Nanotechnology Innovation Center of Kansas State (NICKS), Kansas State University, Manhattan KS, USA

*Corresponding author: Kyoungju Choi

Dr. Kyoungju Choi, Ph.D.

E-mail: kjchoi@ksu.edu

Dr. Hyun Joo, Ph.D.

E-mail: hjoo@ksu.edu

Fax: 785-532-4953

Supplementary Figures

Supplementary Fig. S1

Supplementary Fig. S2

Supplementary Fig. S3

Supplementary Tables

Supplementary Table S1

Supplementary Table S2

Supplementary Fig. S1

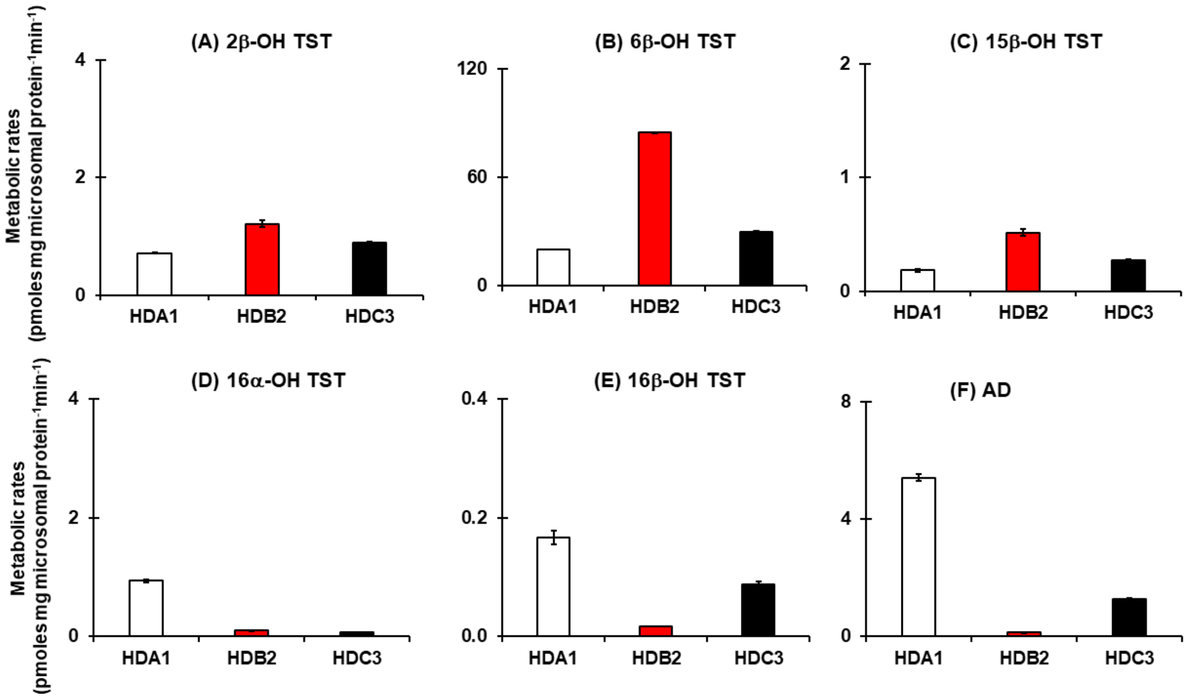

Fig.S1. Production of testosterone metabolites within three different single donor HLM (HDA1, HDB2 and HDC3). Data represent mean ± S.D. (n=3). 2β-OH TST, 2β-hydroxytestosterone; 6β-OH TST, 6β-hydroxytestosterone; 15β-OH TST, 15β-hydroxytestosterone; 16α-OH TST, 16α-hydroxytestosterone; 16β-OH TST, 16β-hydroxytestosterone; AD, androstenedione.

Supplementary Fig. S2

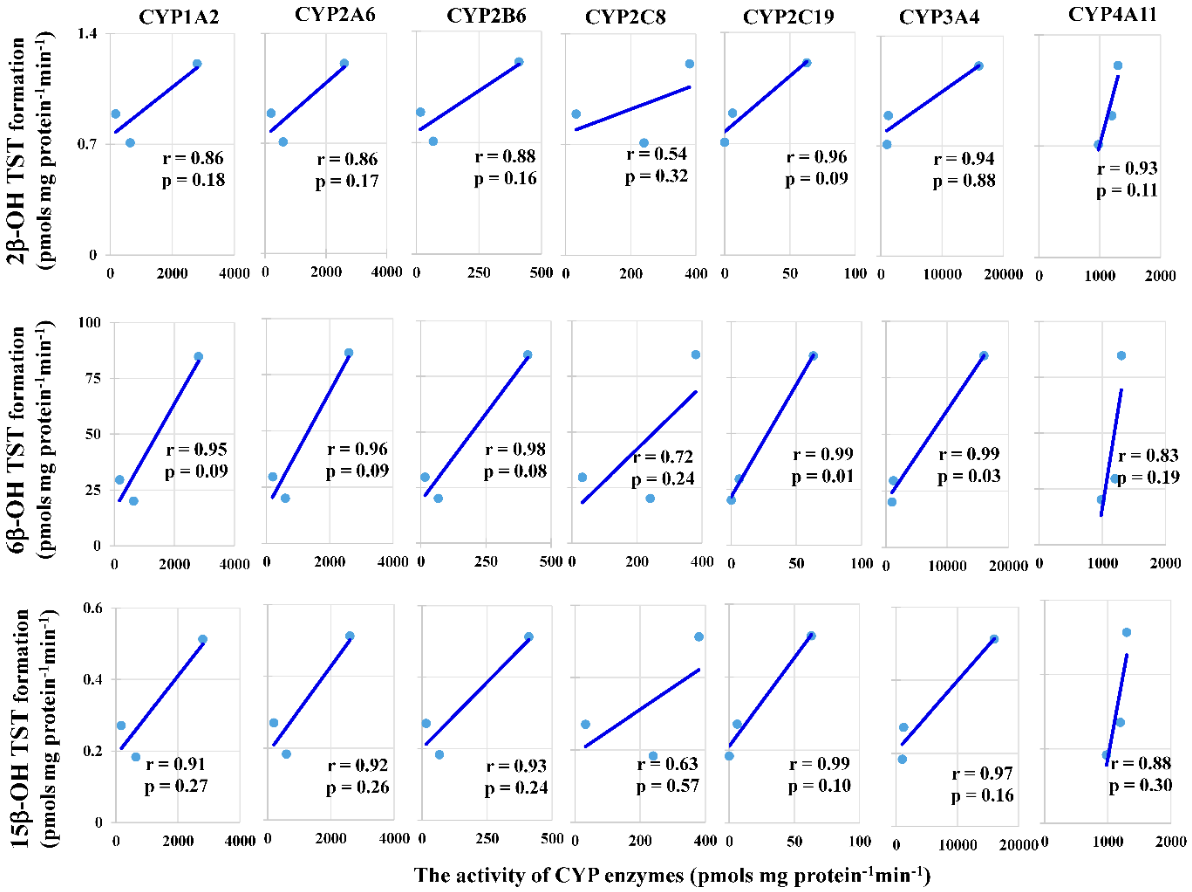

Fig.S2. Pearson correlation coefficient (r) and p-value (p) between CYP activity and the production of TST-derived metabolites in single donor HLM. The catalytic activity of seven CYP enzymes of single donor HLM was adapted from Corning (Table 2). CYP, cytochrome P450; 2β-OH TST, 2β-hydroxytestosterone; 6β-OH TST, 6β-hydroxytestosterone; 15β-OH TST, 15β-hydroxytestosterone; HLM, human liver microsomes.

Supplementary Fig. S3

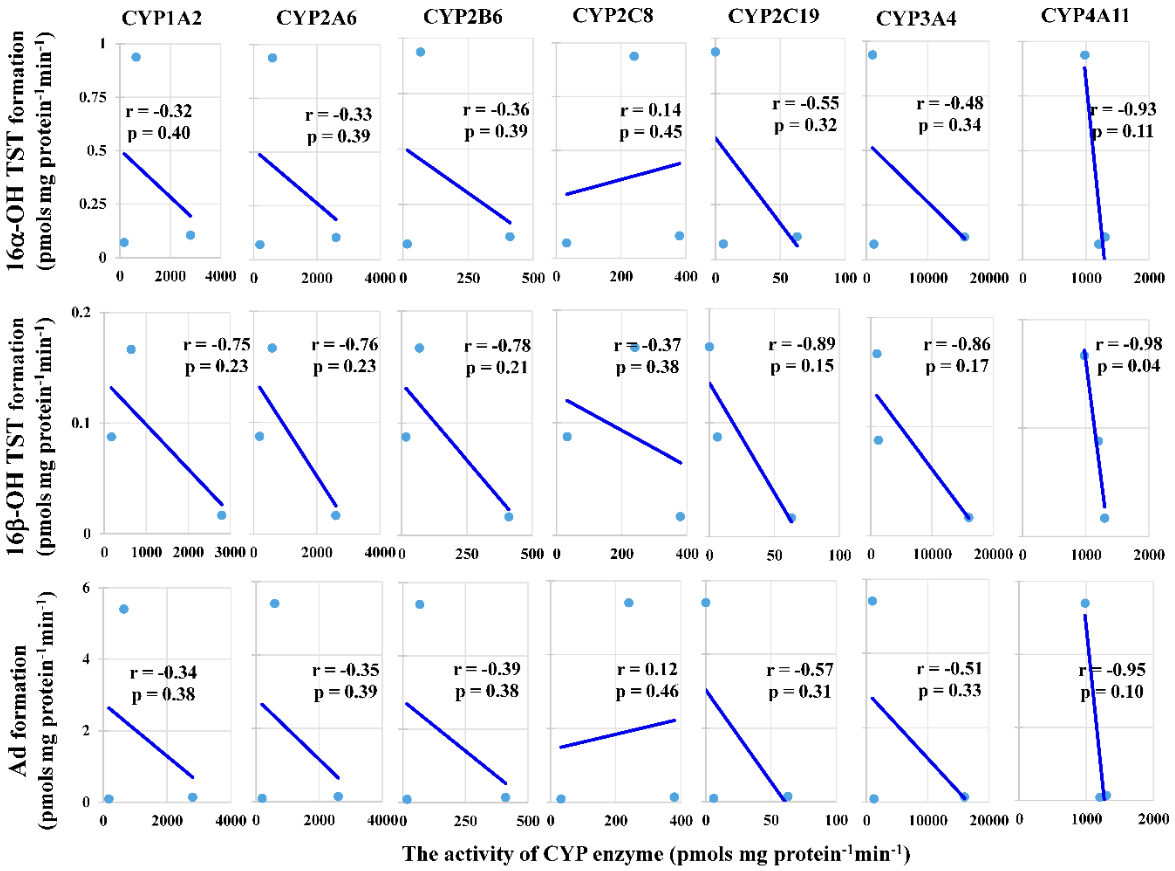

Fig.S3. Pearson correlation coefficient (r) and p-value (p) between CYP activity and the production of TST-derived metabolites in single donor HLM. The catalytic activity of seven CYP enzymes of single donor HLM was adapted from Corning (Table 2). CYP, cytochrome P450; TST, testosterone; 16α-OH TST, 16α-hydroxytestosterone; 16β-OH TST, 16β-hydroxytestosterone; AD, androstenedione; HLM, human liver microsomes.

Supplementary Tables

Supplementary Table S1. Characterization and selected CYP enzyme activity of single donor human liver microsomes

Donor[*]	Race	Age	Gender	Enzyme activity [#]						
				CYP1A2[a]	CYP2A6[b]	CYP2B6[c]	CYP2C8[d]	CYP2C19[e]	CYP3A4[f]	CYP4A11[g]
HDA1	A	55	M	640 (Med)	680 (Med)	67 (Med)	240 (Med)	0 (L)	970 (L)	980 (Med)
HDB2	C	51	F	2800 (H)	2600 (H)	410 (H)	380 (Med)	63 (Med)	16000 (H)	1300 (Med)
HDC3	C	65	M	170 (L)	190 (L)	16 (L)	32 (L)	6.1 (L)	1200 (Med)	1200 (Med)

[*] adapted from Corning high/low P450 single donor human liver microsomes panel; A, Asian; C, Caucasian; F, female; M, male; [#] Respective activities for CYP enzymes presented as pmoles microsomal protein mg^{-1} min^{-1}; H, high CYP activity; Med, median CYP activity; L, low CYP activity; [a] CYP1A2-specific substrate, phenacetin O-deethylase; [b] CYP2A6-specific substrate, coumarin 7-hydrozylase; [c] CYP2B6-specific substrate, (S)-mephenytoin N-demethylase; [d] CYP2C8-specific substrate, paclitaxel 6α-hydroxylase; [e] CYP2C19-specific substrate, (S)-mephenytoin 4-hydroxylase; [f] CYP3A4-specific substrate, testosterone 6β-hydroxylase; [g] CYP4A11-specific substrate, lauric acid 12-hydroxylase

Supplementary Table S2. MRM parameters for determination of testosterone, metabolites and $^{13}C_3$-testosterone (ISTD)

Analytes	Cone voltage (V)	Dwell time (sec)	Parent (m/z)	Product (m/z)	Collision energy (V)	Retention time (min)	Scan time (min)
15β-Hydroxytestosterone	34	0.061	305	97	26	3.71	0.00-4.40
	34	0.061		109	26		
6β-Hydroxytestosterone	26	0.061	305	91	52	4.05	0.00-4.40
	26	0.061		269	14		
16α-Hydroxytestosterone	38	0.028	305	97	26	4.65	4.40-5.25
	38	0.028		109	26		
16β-Hydroxytestosterone	34	0.028	305	97	26	4.95	4.40-5.25
	34	0.028		109	26		
2β-Hydroxytestosterone	28	0.061	305	269	16	5.40	5.25-5.70
	28	0.061		287	16		
Androstenedione	34	0.028	287	97	24	5.95	5.70-8.40
	34	0.028		109	24		
$^{13}C_3$-Testosterone	34	0.028	292	100	24	6.30	5.70-8.40
	34	0.028		112	24		
Testosterone	36	0.028	289	97	24	6.30	5.70-8.40
	36	0.028		109	26		

ISTD, an internal standard
